# Frontal lobe tumor misdiagnose. A case report

**DOI:** 10.1192/j.eurpsy.2022.1638

**Published:** 2022-09-01

**Authors:** A. Franco Soler, H. Torregrosa Martínez, P. Coucheiro Limeres, A. Cerame, P. Prada Bou

**Affiliations:** 1 Hospital Universitario José Germain, Psychiatry Department, Leganés, Spain; 2 Hospital Universitario Príncipe de Asturias, Neurology, Alcala de Henares, Spain; 3 Hospital Universitario José Germain, Hospital De Día, Leganes, Spain; 4 Hospital Universitario Infanta Cristina, Psychiatry, Parla, Spain

**Keywords:** depressive mood, Frontal lobe tumor, craneal tomography

## Abstract

**Introduction:**

Space occupying lesions compromising frontal lobes usually may produce in the first place psychiatric symptoms such as progressive change of personality and/or symptoms suggestive of depressive episodes. Thus they can be misdiagnosed and mistreated.

**Objectives:**

A case report is presented as well as an updated review of frontal lobe tumor diagnosis and treatment literature.

**Methods:**

We present the case of a 45 years-old male patient with no relevant medical history who arrives at the mental health center due to behavioral disorders, depressive mood, workplace absenteeism and personal hygiene neglect in the last 3 months.

**Results:**

Since the clinical picture was compatible with depressive disorder the patient was treated with psychotherapy and antidepressant drugs with no remission. Due to the treatment absence of response he attends emergency services where he is performed a craneal tomograpy (CT) where a right frontal lobe tumor (FLT) is observed.

**Conclusions:**

In early stages FLT are sometimes presented as psychological mood or anxiety disorders without accompanying neurologic deficits. Thus, mental health professionals should be aware that psychological symptoms might be a presentation of organic disease of the brain and in some cases (e.g. middle-aged patients with affective symptoms with no previous mental health history) organic screening and hence brain imaging should be considered.

**Disclosure:**

No significant relationships.

